# Light-Triggered Catalytic Performance Enhancement Using Magnetic Nanomotor Ensembles

**DOI:** 10.34133/2020/6380794

**Published:** 2020-07-08

**Authors:** Fengtong Ji, Ben Wang, Li Zhang

**Affiliations:** ^1^Department of Mechanical and Automation Engineering, The Chinese University of Hong Kong, Shatin N.T., Hong Kong, China; ^2^Chow Yuk Ho Technology Centre for Innovative Medicine, The Chinese University of Hong Kong, Shatin N.T., Hong Kong, China

## Abstract

Micro/nanomachines have attracted extensive attention in the biomedical and environmental fields for realizing functionalities at small scales. However, they have been rarely investigated as active nanocatalysts. Heterogeneous nanocatalysts have exceptional reusability and recyclability, and integration with magnetic materials enables their recovery with minimum loss. Herein, we propose a model active nanocatalyst using magnetic nanomotor ensembles (MNEs) that can degrade contaminants in an aqueous solution with high catalytic performance. MNEs composed of a magnetite core coated with gold nanoparticles as the nanocatalyst can rotate under the action of a programmable external field and carry out rapid reduction of 4-nitrophenol (4-NP). The hydrogen bubbles generated in the catalytic reaction provide random perturbations for the MNEs to travel in the reaction solution, resulting in uniform processing. The reduction can be further boosted by irradiation with near-infrared (NIR) light. Magnetic field induces the rotation of the MNEs and provides microstirring in the catalysis. Light enhances the catalytic activity *via* the photothermal effect. These MNEs are also capable of moving to the targeted region through the application of a programmable magnetic field and then process the contaminant in the targeted region. We expect that such magnetic MNEs may help better in applying active heterogeneous nanocatalysts with magnetic field and light-enhanced performance in industrial applications due to their advantages of low material cost and short reaction time.

## 1. Introduction

Micro/nanomotors have aroused considerable interest in the past decade due to their potential applications in biomedical engineering [1] and environmental remediation [2]. To achieve active control and suitability for use in various environments, external stimuli including magnetic fields [3], light [4, 5], acoustic fields [6], electric fields [7], and chemical reactions [8] are powerful tools for the precise actuation of micro/nanomotors toward the target regions in the preassigned tasks [9]. Although substantial progress has been achieved for improved self-propulsion of micro/nanomotors driven by various chemical reactions, further development of micro/nanomotors is still required to enable their industrial applications. The use of active nanocatalysts may be a useful strategy to achieve this goal for the functional micro/nanomotors. To realize active catalysis with a large amount of tiny motors, motion control of multiple micro/nanomotors is important. Collective behaviors may offer one of the best solutions for multiple agents with high group work efficiency. Colloidal nanomotors can be designed and synthesized as functional agents to conduct tasks [10, 11] or perform patterns [12, 13] collectively. The controllable motion can also be managed by modulating the stimuli on and off [14, 15] or by programming the stimuli [16]. Specifically, the magnetic field is a versatile noncontact approach to realize on-demand transformation [17]. It is programmable for automated control [18], and it is functional for targeted transportation [19], electrical connection [20], and controlling of the plasmonic property of nanocomposites [21].

Magnetic nanomotors can be integrated with designated nanocatalysts for catalytic activity, and they are recoverable in heterogeneous catalysis [22]. Endowed with large specific surface area, catalysts at small scales are promising for promoting catalysis [23, 24]. Particles with magnetic materials, *e.g.*, magnetite (Fe_3_O_4_), can be effective in a wide range of typical catalysis, including the reduction of contaminants [25–27]. However, previous works mainly concentrate on the reusability, and the nanocatalysts are still passive during catalysis. The magnetic nanomotors carrying nanocatalysts can be agents for active catalysis with programmable magnetic field. Moreover, the real time control *via* magnetic actuation is also capable of making nanomotors perform collectively [28]. Such possibilities may promote nanomotors to be active during the whole sequence of contaminants processing, *i.e.*, catalysis, targeted migration, and removal. Specially designed micro/nanomotors are capable of utilizing light to obtain enhanced performance [29]. Owing to the high photon energy of ultraviolet (UV) light, swarming patterns of silica particles can be modulated by controlling the photochemical reaction [30]. Semiconductor particles made of TiO_2_ are useful agents to harvest UV light energy and collectively present phototactic behaviors [31, 32]. Designed with the resonant structure, micro/nanomotors can also absorb near-infrared (NIR) light and generate heat to achieve excellent motion performance [5, 33]. However, the UV light is biohazardous, and NIR light is always of high power required to stimulate micro/nanomotors. Therefore, strategies with harmless light of low power for saving energy are in need of further investigations for industrial applications.

**Figure 1:**
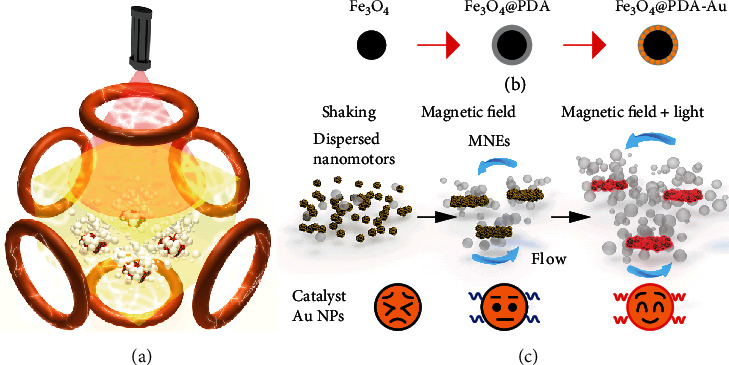
Schematic of MNEs in the catalysts with enhanced performance. (a) MNEs in Helmholtz coils and NIR irradiation for the degradation of 4-NP. (b) Synthesis process of Fe_3_O_4_@PDA–Au nanomotors. (c) Comparison of catalytic performance of MNEs under shaking, magnetic field, and light.

Here, we propose a model of an active nanocatalyst using magnetic nanomotor ensembles (MNEs) to improve the catalytic performance from the combined effects of the magnetic field and irradiation from an NIR laser (Figure 1(a)). The nanomotors acting as the nanocatalyst consist of paramagnetic Fe_3_O_4_ nanoparticles (NPs) coated with gold (Au) NPs on a layer of polydopamine (PDA) (Figure 1(b)). The Au NPs on the nanomotors trigger the reduction of 4-nitrophenol (4-NP) which is used as the model reaction in this study; however, this process is inefficient under mechanical shaking (Figure 1(c)). By contrast, under the application of a magnetic field, the initially dispersed nanomotors aggregate to generate MNEs. These MNEs show a locally increased nanocatalyst concentration and can be actively controlled by the magnetic field to enhance the catalysis. More importantly, the catalytic performance of MNEs can be further boosted by an application of NIR irradiation. As a result, MNEs reach a high catalytic activity under the hybrid stimuli of magnetic field and light, demonstrating their potential industrial impact with regard to the improved catalytic efficiency, shortened catalytic cycle, and cost savings from the reduced noble metal usage.

## 2. Results and Discussion

**Figure 2:**
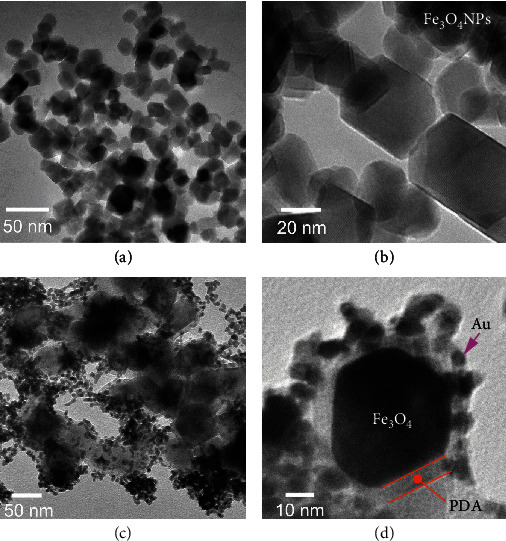
Characterization of magnetic nanomotors. (a) The overview and (b) enlarged view of the TEM images of Fe_3_O_4_ NPs (40 nm). (c) The overview and (d) enlarged view of the TEM images of Fe_3_O_4_@PDA–Au nanomotors with an average size of 50 nm.

The magnetic nanocatalysts are composed of a Fe_3_O_4_ core with an average diameter of 40 nm, a PDA shell, and a surface layer of densely arranged Au nanocrystals. Transmission electron microscopy (TEM) images presented in Figures 2(a) and 2(b) show the overall view and an enlarged view of the Fe_3_O_4_ NPs with excellent crystallinity, obtained by the hydrothermal treatment of the Fe_3_O_4_ NPs synthesized by coprecipitation. As is known, PDA is an effective adhesion layer that works in liquid environment [34]. To increase the coating stability of Au NPs on the nanomotors, we use a PDA layer to fix and anchor the Au NPs. After coating with the PDA shell, the Fe_3_O_4_ NPs are further coated with Au nanocrystals with an average size of 5 nm by an electrostatic force (Figures 2(c) and 2(d)). The Au NPs on the nanomotors are quite stable by our proposed method (Materials and Methods), and they remain attached to the surface because of the PDA layer. These nanomotors loaded with Au NPs are initially dispersed when added into the reaction solution and then form stripe-like microensembles due to the particle-particle interactions between their paramagnetic Fe_3_O_4_ cores under an application of the magnetic field. Because of the carried Au NPs, these MNEs act to degrade 4-NP in an aqueous solution in the presence of NaBH_4_.

MNEs with a locally high concentration due to the aggregation effect of the nanomotors effectively promote catalysis with microstirring due to the application of a rotating magnetic field and the photothermal effect on the solution due to light irradiation (Figure S1). Here, we use the catalytic conversion of 4-NP to 4-aminophenol (4-AP) as an example. As a toxic organic compound with high solubility, the 4-NP is commonly found in industrial effluents due to its broad application in pesticides, dyes, and drugs [35]. Therefore, the degradation of 4-NP prior to its release into the environment is of great importance. 4-NP shows high stability in the natural environment, and the conversion of the 4-NP to 4-AP may require a suitable catalyst with low cost and high efficiency. Magnetic nanomotors carry Au NPs that trigger the reduction of 4-NP, and the large specific surface area of these NPs is beneficial for their catalytic performance. Initially, the solution was yellow due to the presence of 4-NP, and the reduction in the 4-NP amount was accompanied by discoloration as colorless 4-AP was produced (Movie S1). Under the existence of the nanocatalysts, the NaBH_4_ reacts with water rapidly and forms the reductive BH_4_^–^ and H_2_ bubbles [23]. These two kinds of reductive products can finally convert the 4-NP to 4-AP. The bubbles are growing as the catalysis progresses, especially when magnetic field and light are both on (Figure S2). These bubbles provide random perturbations to MNEs for improving the catalysis.

**Figure 3:**
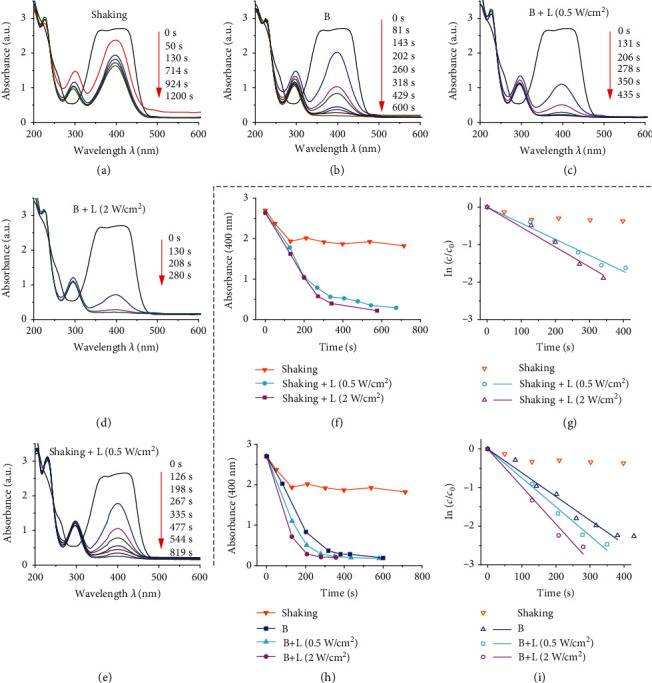
Degradation of 4-NP using MNEs. (a) Absorption spectra of processed 4-NP solution obtained at different times under shaking on a shaker at 300 rpm as the control group, (b) the application of a magnetic field (B) only, (c) a magnetic field and light (L) with the power density of 0.5 W*/*cm^2^, (d) a magnetic field and light with the power density of 2 W*/*cm^2^, and (e) light with the power density of 0.5 W*/*cm^2^ and shaking. (f) Absorption peaks and (g) ln(*c*/*c*_0_) as a function of time for the processing under light. (h) Absorption peaks and (i) ln(*c*/*c*_0_) as a function of time for the processing under the integrated application of magnetic field and light. The slopes of the linear fitting lines indicate the reaction rates, and a larger slope implies a faster reaction. The magnetic field strength and frequency were 4 mT and 10 Hz, respectively. The *c* and *c*_0_ represent the concentrations of 4-NP at time *t* and 0 s.

The high catalytic performance of MNEs under magnetic field and light was quantitatively evaluated by ultraviolet-visible (UV-vis) spectroscopy. As shown in Figure 3(a), the intensity of the characteristic absorption peak of 4-NP at 400 nm decreases, indicating the decrease in the concentration of 4-NP due to its degradation over time. The sample is placed on a shaking plate operated at a rate of 300 rpm to simulate the common industrial environment and macroscopic disturbances, and the nanomotors disperse homogeneously in the solution. However, the degradation ends prior to the full degradation of 4-NP because the nanocatalyst concentration is too low to continue the reduction after the 4-NP concentration becomes sufficiently low. By contrast, the peak almost vanishes at 600 s for MNEs catalysis under magnetic field only (Figure 3(b)). The locally high concentration of nanocatalyst maintained by MNEs ensures the effective catalysis and accomplishes the complete reduction of 4-NP. Furthermore, under illumination (Figures 3(c) and 3(d)), the absorption peak intensity decreases rapidly, and the spectrum becomes almost flat after irradiation for 280 s by light with an intensity of 2 W*/*cm^2^. Thus, the processing time is further reduced by 16% compared to the application of the magnetic field only. In order to demonstrate the effect of light, the catalytic performance of nanomotors was tested under shaking and light (Figure 3(e)). It was found that irradiation at a low power density of 0.5 W*/*cm^2^, which is harmless and permissible for skin exposure [36], can overcome the concentration barrier and accelerate the reduction.

The catalytic enhancement of MNEs results from both magnetic actuation and irradiation. The irradiation enhances the interaction between the reactants and MNEs. The catalytic activity is enhanced as the light power density increases to 2 W*/*cm^2^ (Figure 3(f)). For a quadrupling of the light intensity, the reaction rate is increased by 24% (Figure 3(g) and Figure S3), indicating a trade-off between energy and efficiency. By further integrating light irradiation with an application of a weak magnetic field at a low energy cost, very rapid catalytic degradation can be achieved (Figure 3(h)). Under an application of the magnetic field only, catalysis can be clearly accelerated with a rate as fast as 144% of the reaction rate obtained at the irradiation with a power density of 0.5 W*/*cm^2^ and shaking (Figure 3(i)). Based on the magnetic actuation, the reaction rate can be further increased by 22% and 48% for the irradiation intensities of 0.5 W*/*cm^2^ and 2 W*/*cm^2^, respectively. In a rotating magnetic field, MNEs can retain a high local nanocatalyst concentration with a small initial dose (Materials and Methods), in order to continue the catalysis unlike for the processing in the violent shaking condition. During the catalysis, the magnetic field drags MNEs to induce rotation, resulting in stirring on a small scale. Irradiation enhances the interactions between the reactants and the nanocatalyst. Moreover, the combination of rotation and unstable hydrogen bubbles also leads to random motion of MNEs and their spreading in the tank. The magnetic agitation and photothermal enhancement are thus integrated to maintain the high catalytic performance of the MNEs. Such enhancement is helpful for industrial applications due to the short time and low materials cost of this process.

**Figure 4:**
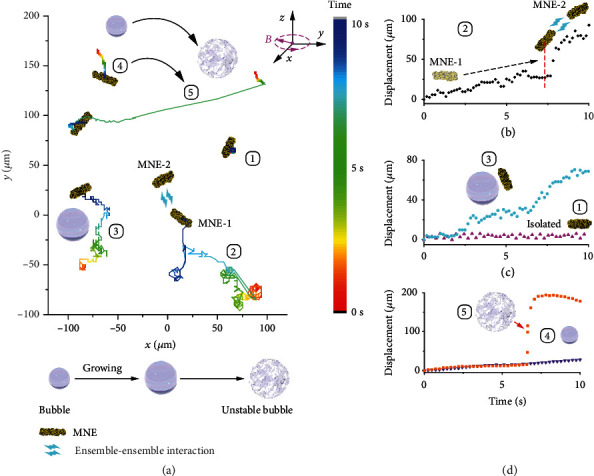
Representative motion of the MNEs during catalysis. (a) Typical trajectories of the MNEs for the cases of an isolated MNE (Trajectory 1), MNE near another MNE (Trajectory 2), MNE near a growing bubble (Trajectory 3), MNE at a distance of approximately 600 *μ*m to a growing bubble (Trajectory 4), and MNE near an unstable bubble (Trajectory 5). The color scale shows the time of the bubble location in the trajectories. The inset shows a rotating magnetic field. (b) The displacement of the MNEs for the case of ensemble-ensemble interaction, (c) MNE rotating near a bubble and isolation, and (d) MNE rotating at a large distance from a growing and unstable bubble. The red dashed line in (b) indicates the position at the critical distance between the two MNEs.

Growing bubbles introduce random perturbations to induce the rotating MNEs to travel randomly and achieve uniform catalysis (Movie S2). Some representative types of motion are presented in the illustration of the ongoing degradation of 4-NP (Figure 4(a)). Due to sedimentation, the isolated MNEs, *e.g.*, those far away from the bubbles or other MNEs, may rotate almost in place under a rotating magnetic field (Trajectory 1). When MNEs are in close proximity, which is a common phenomenon at the high concentration of magnetic nanomotors, they are attracted to each other and merge into a larger MNE due to their ensemble-ensemble interactions (Trajectory 2). One MNE performs directional motion toward the other MNE at first. Then, when the MNEs reach the critical separation distance, the ensemble-ensemble interaction dominates and the MNEs rapidly collide. Therefore, the displacement of the MNE clearly increases (Figure 4(b)). Moreover, when an MNE rotates in the vicinity of a growing bubble, its motion can be influenced by the tension from the liquid-air interface (Trajectory 3). The MNE moves along the bubble, and the translational motion is directed by the expanding bubble (Figure 4(c)). By contrast, for an MNE located at a larger distance from a bubble, the growing bubble has almost no influence on its motion (Trajectory 4). However, when the bubble grows sufficiently to become unstable and exhibits a stronger perturbation, the MNE shows a high displacement in a short time (Trajectory 5). The continuous generation of such unstable bubbles leads to the fast translational motion of the MNEs (Figure 4(d)). Such perturbations can enhance the mass exchange and the mobility of nanomotor ensembles in the reaction by introducing active convection. Therefore, as a result of the integration of these types of motion, MNEs travel in the reaction solution and promote catalysis.

**Figure 5:**
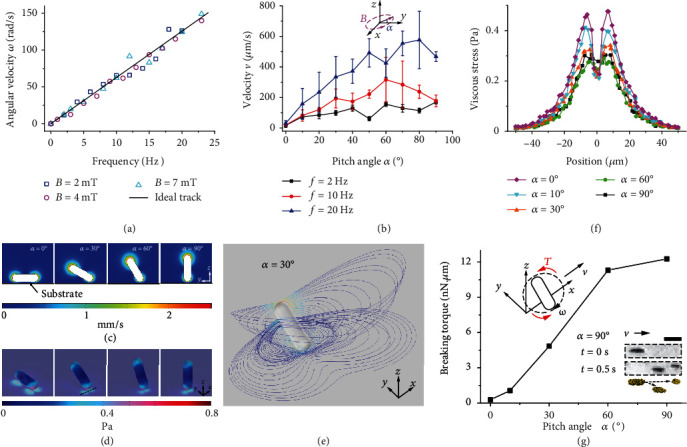
Interactions with boundaries of rotating MNEs. (a) Frequency response of the MNEs under magnetic fields with different strengths. (b) Velocity of the MNEs under magnetic fields with different pitch angles. The magnetic flux density was 4 mT, and the error bar shows the standard error of the velocities obtained from five samples. The inset shows a rotating magnetic field with a pitch angle *α*. (c) Velocity fields around pitched MNEs. The color scale indicates the magnitude of the fluid velocity. (d) The viscous stress distribution on the surface of the pitched MNEs and the substrate. The color scale indicates the magnitude of the viscous stress. (e) Streamlines near the MNE (Materials and Methods). The color of the streamlines indicates the fluid velocity, and the color scale is same as that in (c). (f) The viscous stress distribution along the line on the substrate in the forward +*x* direction. This line is the dashed line shown in (d), which is the projection of the nearest boundary of the MNE on the substrate during rotation. (g) Breaking torque exerted on the MNEs. The upper inset illustrates the breaking torque *T*. The lower inset shows an MNE breaking into two fragments during translational motion in (b) at 10 Hz, and the bar corresponds to 50 *μ*m. Data in (c)–(g) are obtained from the simulation based on the experiments in (a) and (b).

The motion of MNEs makes them active nanocatalysts in catalytic reactions. Comparing the angular velocity of MNEs and the frequency of magnetic field, the tracking of rotating MNEs shows that they exhibit a strong response to the external field (Figure 5(a)). This may enable the control of the reaction rate through the regulation of the magnetic field. A magnetic field of 2 mT can drive the MNEs to carry out full degradation of 4-NP in the aqueous solution at a high field frequency of 25 Hz without losing tracking to the external field. Thus, the dose of the nanomotors can remain small. In addition to the rotation, directional motion of MNEs can also be achieved for targeted catalysis by adding a pitch angle *α* to the rotating plane (Figure 5(b)). While the application of a high frequency (20 Hz) magnetic field results in a high angular velocity of MNEs, the pitch angle is critical for enabling the MNEs to move stably and reach the region targeted for catalysis. It is preferred to maintain a small velocity fluctuation and stable structure in the migration of a group of MNEs.

Both in catalysis and migration, MNEs interact with the fluid and the substrate. The interaction between the MNEs and the fluid dominates the catalysis, while the interaction with the substrate determines the mobility of the MNEs. Such interactions induced by the viscous flow provide the driving for the reaction, and balance the driving force due to magnetic actuation. However, such a strong interaction may break the MNEs into small fragments or even nanomotors, resulting in the loss of the nanocatalyst. When the MNEs aggregate in preparation for migration, they merge into larger MNEs for effective directional motion (Figure S4). Herein, we model the MNE as a round-head cylinder with a length and radius of 70 *μ*m and 10 *μ*m, respectively, and show the local fluid environment in viscous flow induced by the boundaries. A crevice with the size of 5 *μ*m is assumed at a constant distance to accommodate the viscous flow. When an MNE is pitched by the magnetic field on the substrate, the fluid is almost static near the lower end because of the nonslip boundary (Figure 5(c)). Correspondingly, a 3D view shows that the maximum stress is found at the lower end of the MNE and reaches 0.8 Pa (Figure 5(d)). The lower end is confined by the substrate, while the upper end rolls forward freely. Thus, the asymmetric viscous flow results in the directional displacement of the MNEs in each cycle. Between the MNE and substrate, circular flows are induced by the rotating MNEs used for the catalysis. In a pitched rotation, the stream pattern of these circular flows is concave downward below the MNE and rises above the MNE (Figure 5(e)). Due to such rising flows, the pitched MNE may deviate slightly from the preassigned direction when rotating on the substrate.

The viscous stress on the surfaces determines the directional motion and whether the MNEs break during this time period. The substrate that provides the boundary condition and reaction for MNEs withstands the stress with double peaks when the lower end of MNE is closest to the substrate (Figure 5(f)). The magnitude of the stress decreases with the increasing pitch angle, but shows almost no difference for the pitch angles greater than 30°. This is because with the exception of the effect from the confined flow in the small crevice, the fluid beneath the rotating plane undergoes almost no extrusion at large pitch angles. To simplify the analysis, the torque due to the axial dimension of the MNE can be neglected, and the torque for the MNEs rotating in *yz* plane is too small to be effective (Figure S5). However, the torque due to the stresses along the *x*-axis (Materials and Methods) may break the MNE into two or more fragments, and loss of MNEs may occur in this case (Figure 5(g)). The breaking torque becomes stronger as the pitch angle increases; therefore, a smaller pitch angle is preferable to maintain unbroken and steadily moving MNEs.

It is vital for the MNEs to remain as a group to guarantee the successful migration of the amount of MNEs necessary for targeted catalysis and maintain the catalytic ability. Generally, not all nanomotors can form MNEs, and some may remain dispersed in the solution. Dispersed nanomotors may be left or lost during the directional motion due to their weak magnetic response and obstacles encountered during transport, *e.g.*, the bubbles in this catalytic reaction. While it is difficult to move these MNEs, they can still be recycled by a magnet. Strong interactions with the boundaries may break the MNEs and result in a loss of the nanomotors during migration. Therefore, the pitch angle for the motion should be controlled to remain small in order to decrease the breaking torque exerted on the MNEs and retain them as a compact group. Such a controllable motion of the MNEs enables them to reach the targeted regions or the recycling site at which a permanent magnet or an electromagnet can be placed.

**Figure 6:**
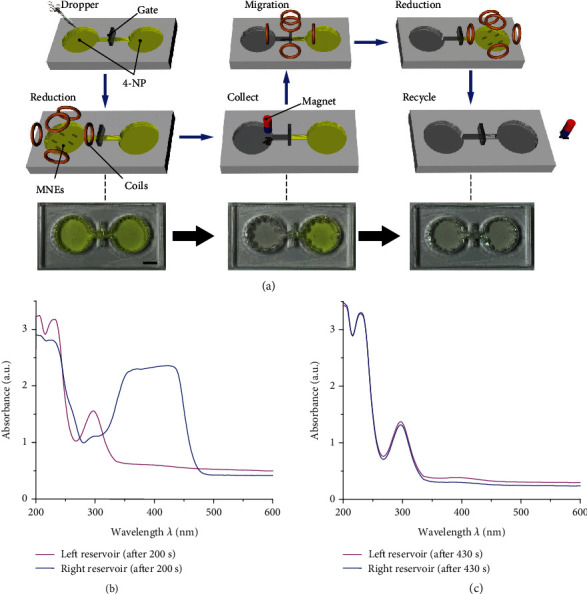
Reusable and recyclable MNEs. (a) Sequential degradation of 4-NP in left and right reservoirs. The scale bar is 5 mm. (b) UV-vis absorption spectra of the solution in left and right reservoirs after 200 s and (c) when the reduction sequence is completed in 430 s.

MNEs can be reused for targeted catalysis and recycled by easy magnetic removal. In separate reservoirs, they can process the contaminants in a sequence (Figure 6(a)). The left and right reservoirs are connected through a channel that can be controlled to be either open or closed by a glass gate. The channel is initially closed. During the separate processing of reservoirs, each reservoir is set in the center of the workspace (18 mm × 18 mm × 18 mm) in Helmholtz coils, where the magnetic field is uniform. First, nanomotors are dropped into the left reservoir. The Helmholtz coils generate the rotating magnetic field, and then the nanomotors form the MNEs. These MNEs rotate with the field and trigger the catalytic reaction. After 200 s, the 4-NP in the left reservoir is degraded completely, while a high concentration of 4-NP is still present in the right reservoir (Figure 6(b)) due to the blocking of diffusion by the glass gate. MNEs are collected in the solution with the assistance of a permanent magnet for migration in the next step. Then, after the glass gate is removed, a pitched rotating magnetic field can be used to move the MNEs through a channel and reach the right contaminated reservoir after approximately 30 s. After closing the glass gate, the rotating field is reactivated for the reduction in the right reservoir, and the solution is fully processed after 200 s (Figure 6(c)). Since the carried Au NPs catalyst is not consumed during the catalytic reaction, MNEs can be reused for the degradation of the contaminants in the targeted location. Finally, MNEs can be recycled by a magnet, and the environmental remediation catalysis is thus completed.

## 3. Conclusion

In summary, we have demonstrated a model of MNEs as a recoverable nanocatalyst showing magnetic field and light enhanced catalytic performance. The MNEs consist of Fe_3_O_4_@PDA–Au nanomotors, and these nanomotors form the MNEs under the application of a magnetic field. The Au NPs coated on the surface of the nanomotors trigger the catalysis, and MNEs with the locally high concentration of the nanomotors display high catalytic performance in a rotating magnetic field. The irradiation by NIR light can break the concentration barrier and further boost the catalysis. With a small dose of 10 *μ*g*/*mL of nanomotors, degradation of 4-NP can be accomplished rapidly after 280 s through the application of a rotating magnetic field and light irradiation. The introduction of NIR light can further reduce the degradation time by 16% compared to the processing with magnetic field only. Harmless irradiation with the intensity of 0.5 W*/*cm^2^ provides clear enhancement of the catalysis. MNEs also undergo directional motion under the action of the programmable magnetic field and reduce the concentration of the 4-NP contaminant in the targeted region. The MNEs are reusable, mobile, and recyclable, realizing active and environmental catalysis. Therefore, catalytic reactions, particularly those triggered by Au NPs, can be conducted more effectively following the approach of this study. Our analysis may provide better understanding to exploit the functionalities of magnetic nanomotors. It can motivate investigation of methods for conducting catalysis with on-demand control, and broaden the environmental and industrial applications of active heterogeneous catalysts. We also anticipate that this study can provide a paradigm for the future design of magnetic plasmonic nanocatalysts with extraordinary catalytic performance.

## 4. Materials and Methods

### 4.1. Materials

Ferrous chloride tetrahydrate, ferric chloride hexahydrate, ammonia solution (25%–28%), sodium borohydride, 4-NP, citrate sodium, and gold chloride trihydrate were purchased from Aladdin Chemical Co., Ltd. (Shanghai, China). Tris(hydroxymethyl)aminomethane was purchased from Acros Organics. All of the chemicals are analytical reagents and are used as received without further purification.

### 4.2. Preparation of Fe_3_O_4_ NPs (40 nm)

The preparation of Fe_3_O_4_ NPs was conducted following the modified coprecipitation method [37]. Briefly, FeCl_3_·6H_2_O (1 M, 10 mL) was mixed with FeCl_2_·4H_2_O (1 M, 5 mL) in a flask under continuous N_2_ flow at 70°C. The mixture was mechanically stirred, and concentrated ammonia (20 mL) was added dropwise to the solution. After the reaction, the black suspension was transferred and sealed in a Teflon-lined stainless-steel autoclave and heated at 250°C for 24 h. After the autoclave was cooled to room temperature, the black products were washed three times with ethanol and deionized (DI) water, respectively. The magnetic particles were finally stored in DI water for further usage.

### 4.3. Synthesis of Fe_3_O_4_@PDA NPs

First, we prepared a 10 mM aqueous solution of tris(hydroxymethyl)aminomethane and adjusted the pH of the solution to 8.5, forming the Tri-HCl buffer. Second, Fe_3_O_4_ NPs (0.1 g) was dispersed in the buffer with ultrasonic treatment for half an hour. Third, dopamine powder (0.02 g) was poured into the solution, and a continuous ultrasound treatment was applied for 5 hours in an ice-water bath. Finally, the black product was collected with a magnet, followed by rinsing several times with DI water and ethanol to remove the surface impurities. The Fe_3_O_4_@PDA NPs were stored in DI water with a concentration of 1 mg*/*mL for further usage.

### 4.4. Synthesis of Au NPs

The synthesis of Au NPs was carried according to the previous studies [38, 39]. Briefly, DI water (60 mL) was added into a bottom-round flask with magnetic stirring in the dark. Then, tiny amounts of HAuCl_4_·3H_2_O solution (600 *μ*L of 25 mM) and sodium citrate (600 *μ*L of 25 mM) were added into the flask. After 10 min, 900 *μ*L 0.2 M NaBH_4_ solution was added into the flask. The color of the solution immediately changed to wine red. After continuous stirring for another 15 min, the solution was aged for 2 hours in the dark prior to the next process.

### 4.5. Synthesis of Fe_3_O_4_@PDA–Au Nanomotors

First, citrate buffer (50 mL) with pH = 2 was prepared (HCl was used to adjust the pH). Second, Fe_3_O_4_@PDA NPs (10 mg) was dispersed in a citrate buffer and sonicated for 10 min. Third, the colloidal solution of Au NPs was rapidly added to the Fe_3_O_4_@PDA NPs suspension with the sonication for 5 min. The resultant magnetic material was collected using a magnet and rinsed three times with DI water. The as-prepared Fe_3_O_4_@PDA–Au nanomotors were stored in the dark at 4°C in DI water with a concentration of 1 mg*/*mL for magnetic actuation and catalysis.

### 4.6. Catalysis of 4-NP by Fe_3_O_4_@PDA–Au MNEs under Different External Stimuli

First, the mixture of 5 mM 4-NP aqueous solution (200 *μ*L) and ice-cooled NaBH_4_ (2 mL) aqueous solution (0.2 M) were prepared in a glass vessel with shaking. The resultant solution was quickly subjected to UV-vis characterization, and the obtained spectrum was assigned to the reaction starting point (*t* = 0*s*). Then, Fe_3_O_4_@PDA–Au nanomotors solution (1 mg*/*mL, 20 *μ*L) was added into the vessel. The vessel was subjected to the following four modes of external stimuli: mechanical shaking, magnetic field, light irradiation with shaking, and magnetic field plus light irradiation (Figure 3). The testing sample was 200 *μ*L of the solution. The UV-vis spectra were obtained sequentially at different points in time in the wavelength range of 200 ∼ 600 nm. The scanning speed was 2400 nm*/*min.

The catalysis by reusable and recyclable MNEs was carried out in two reservoirs (Figure 6). The platform with two reservoirs was fabricated by laser cutting. The body of the tank was made of poly(methyl methacrylate) (PMMA) with a thickness of 3 mm. This PMMA framework was glued on a glass slide. The diameter of each reservoir is 12 mm, and the channel is 6 mm in length and 2 mm in width. First, NaBH_4_ (0.2 M, 200 *μ*L) and 4-NP (5 mM, 20 *μ*L) were added to each reservoir. A glass gate was then placed in the middle of the channel to avoid diffusion between the two reservoirs. A nanomotors solution (2 mg*/*mL, 20 *μ*L) was added to the left reservoir. A rotating magnetic field with a strength and frequency of 5 mT and 12 Hz, respectively, was generated to process the solution in the left reservoir for 200 s. Then, MNEs were collected using a magnet over the solution, and they were regulated to enter the channel by adjusting the magnetic field [19] under the observation from the top view. Afterwards, the magnetic field was set with a pitch angle of 30° for migration to the right reservoir. The migration of the MNEs lasted for 30s, and then the glass gate was closed. The magnetic field was programmed as the rotating field with no pitch angle, and MNEs processed the solution in the right reservoir for another 200 s.

### 4.7. Characterization

Transmission electron microscopy (TEM) images were obtained using a Tecnai F20 system (FEI, USA). UV-vis spectra were obtained using a U2910 UV-vis spectrophotometer (Hitachi, Japan) with two kinds of beams. Image analysis was conducted using Fiji ImageJ.

### 4.8. Numerical Simulation of Moving MNEs

The numerical simulation was conducted using COMSOL Multiphysics. To simplify the analysis, the MNE is modeled as a cylinder with round ends. The nearest distance to the substrate during rotation is set as 5 *μ*m for illustration. The MNE rotates at a frequency of 10 Hz and the translational velocity is based on the experimental data presented in Figure 5(b). The momentum equation in this model follows [40] and is given by

(1) $$\begin{array}{c} \rho \frac{\partial u}{\partial t}=F+\nabla \cdot \left(-pI+µ\left(\nabla u+{\left(\nabla u\right)}^{\text{T}}\right)\right), \end{array}$$

where *ρ* is the density of the fluid, **u** is the flow velocity, **F** is the body force, *p* is the pressure, **I** is the identity tensor, and *μ* is the dynamic viscosity. The streamlines (Figure 5(e)) start from 55 points, and these points are evenly distributed on a line segment coordinated with −30*μ*m ≤ *x* ≤ 25*μ*m, −30*μ*m ≤ *y* ≤ 25*μ*m, and 0*μ*m ≤ *z* ≤ 55*μ*m. The geometry center of the MNE is located at a distance of 0.5 *μ*m from the origin in the +*x* direction. The magnitude of the stress of the substrate is the norm of the stresses over the substrate (Figure 5(f)). The torque on the MNE is the integrated surface stress on the MNE boundaries multiplied by the distance to the geometry center over the whole MNE surface (Figure 5(g) and Figure S5 ).

## Data Availability

All of the data that support the findings of this study are available from the authors upon reasonable request.

## Supplementary Materials

Supplementary Materials Figure S1: NIR absorption spectra of the reaction solution. Figure S2: growing bubbles. Figure S3: UV-vis absorption spectra of the 4-NP solution with time under shaking and strong irradiation. Figure S4: directional motion of MNEs with different pitch angles. Figure S5: torque exerted on the MNE in the *yz* plane as a function of the pitch angle. Movie S1: discoloration of the reaction solution with 4-NP degraded by the MNEs under a magnetic field and light irradiation. Movie S2: representative motion of the MNEs during the catalysis.
