# Supplementary material for: Light-Triggered Catalytic Performance Enhancement Using Magnetic Nanomotor Ensembles
Source: Research (Wash D C). 2020 Jul 8;2020:6380794. doi: 10.34133/2020/6380794 (PMC7366293; doi:10.34133/2020/6380794)
Supplement: Supplementary Materials — Figure S1: NIR absorption spectra of the reaction solution. Figure S2: growing bubbles. Figure S3: UV-vis absorption spectra of the 4-NP solution with time under shaking and strong irradiation. Figure S4: directional motion of MNEs with different pitch angles. Figure S5: torque exerted on the MNE in the yz plane as a function of the pitch angle. Movie S1: discoloration of the reaction solution with 4-NP degraded by the MNEs under a magnetic field and light irradiation. Movie S2: representative motion of the MNEs during the catalysis. [file 6380794.f1.zip › Ji_Supporting_Information_revised.pdf]

Supporting Information:
Light-Triggered Catalytic Performance Enhancement Using Magnetic Nanomotor Ensembles

Fengtong Ji[1], Ben Wang[1], and Li Zhang[1,2*]

[1]Department of Mechanical and Automation Engineering,

The Chinese University of Hong Kong, Shatin N.T., Hong Kong, China.

[2]Chow Yuk Ho Technology Centre for Innovative Medicine,

The Chinese University of Hong Kong, Shatin N.T., Hong Kong, China.

*Correspondence should be addressed to Li Zhang; lizhang@mae.cuhk.edu.hk

1 Supporting Figures

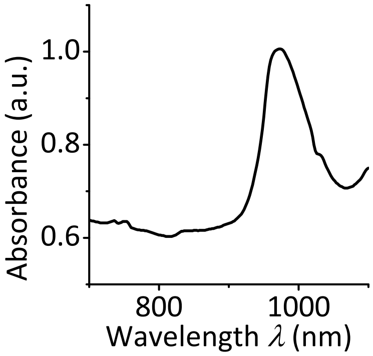

Figure S1: NIR absorption spectra of the reaction solution.

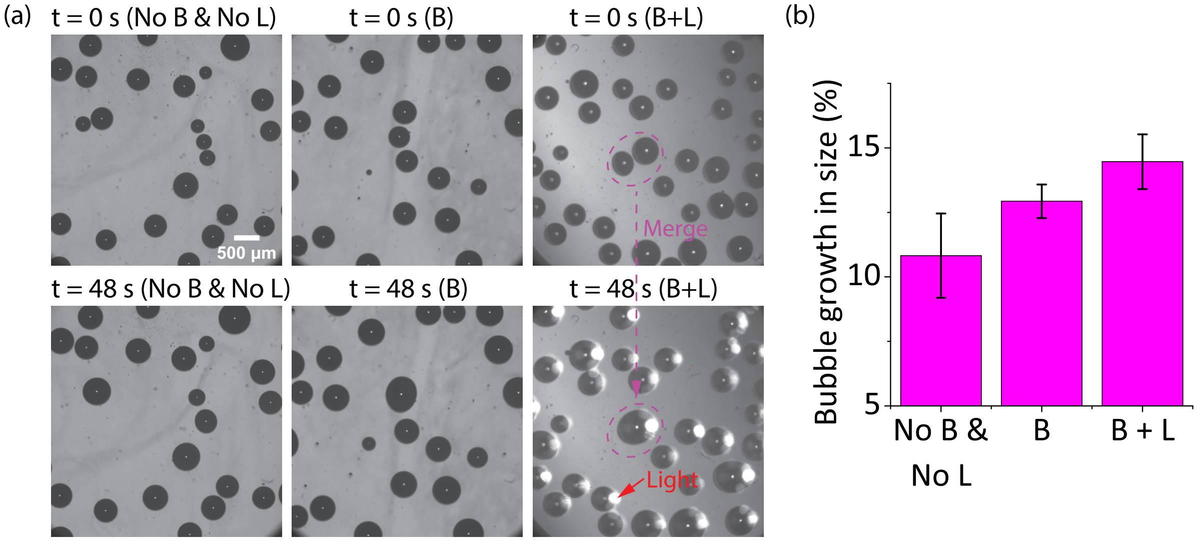

Figure S2: Growing bubbles. (a) Bubbles growing on the glass substrate indicate the reaction rate. A side laser irradiates the right half of the bubbles for illustration. (b) Bubble growth in the catalytic reaction under different stimuli. The initial states of the bubbles in these three conditions are controlled to be about the same based on the average bubble size in the field of view, *i.e.*, with a radius around $252\,\mu\mathrm{m}$. The bubble growth is evaluated in diameter, and error bars indicate the standard deviation of five growing bubbles under each condition.

Description of Figure S2. The experiment on the bubbles was conducted in an open tank ($16\,\mathrm{mm} \times 16\,\mathrm{mm} \times 3\,\mathrm{mm}$) with a glass substrate. The mixture of $NaBH_4$ ($0.2\,\mathrm{M}$, $3\,\mathrm{mL}$) and 4-NP ($5\,\mathrm{mM}$, $200\,\mathrm{\mu L}$) was prepared in the tank. The nanomotor solution ($1\,\mathrm{mg/mL}$, $20\,\mathrm{\mu L}$) was then added into the solution. The magnetic field strength and frequency were $4\,\mathrm{mT}$ and $5\,\mathrm{Hz}$, respectively. The lateral irradiation power density was $1.5\,\mathrm{W/cm^2}$, and the laser beam was transmitted through a 2-mm-thick poly(methyl methacrylate) wall of the tank to illuminate the reaction solution.

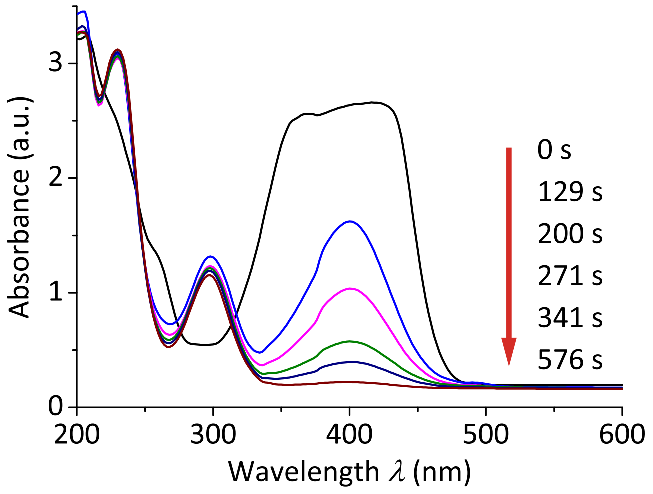

Figure S3: UV-vis absorption spectra of 4-NP solution with time under shaking and strong irradiation. The light power density is $2\,\mathrm{W/cm^2}$.

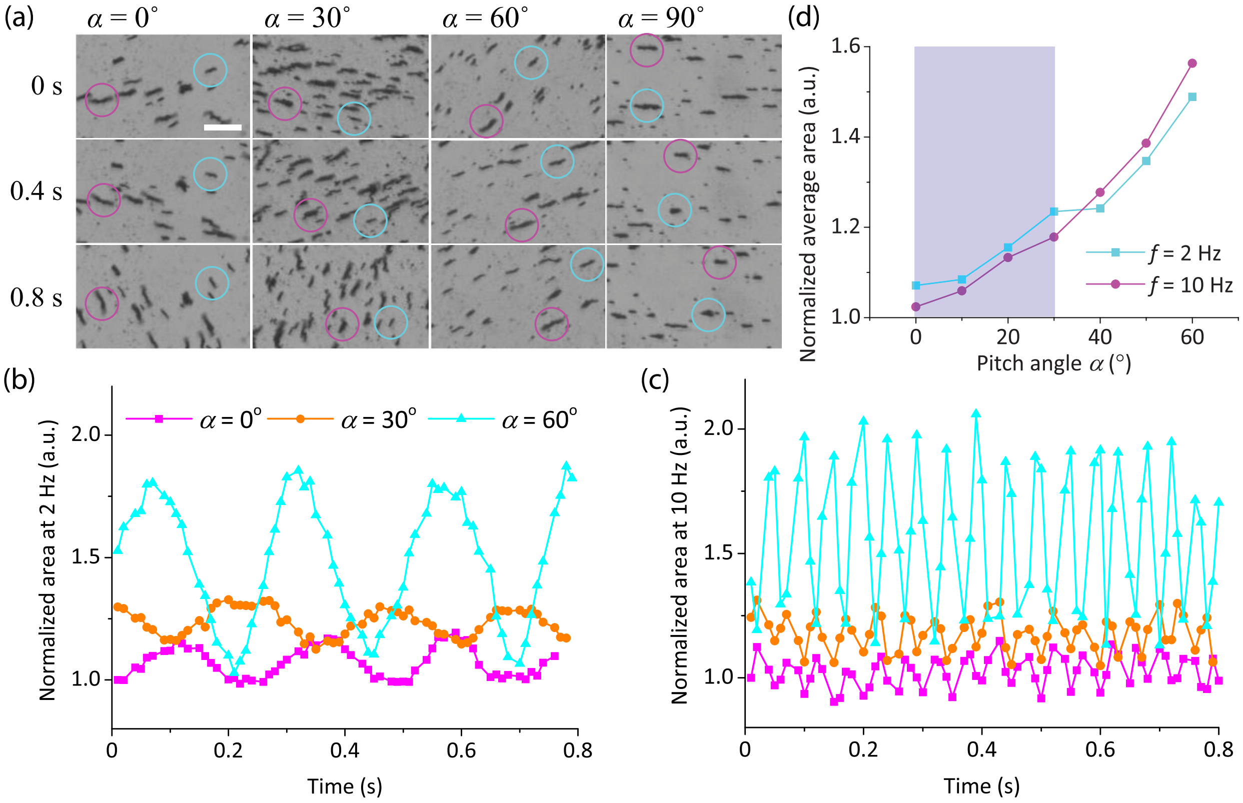

Figure S4: Directional motion of MNEs with different pitch angles. (a) The time-lapse images of MNEs with different pitch angles. The circles with the same color indicate the positions of the same MNE at different times in each column. The scale bar is $100\,\mu\mathrm{m}$. (b) Normalized area of MNEs from top view as a function of time at $2\,\mathrm{Hz}$ and (c) $10\,\mathrm{Hz}$. Data in (b) and (c) share the same legend as those in (b). (d) Normalized average area of MNEs as a function of the pitch angle at $2\,\mathrm{Hz}$ and $10\,\mathrm{Hz}$.

Description of Figure S4. MNEs can conduct directional motion under a pitched magnetic field. While a rotating magnetic field may retain MNEs rotating in place, pitched rotation results in the directional migration of the MNEs (Figure S4(a)). Dispersed nanomotors are not sufficiently dense to conduct stable migration, therefore, retaining compact MNEs is critical. The normalized area which is the area of the MNEs normalized by the initial area is used here to estimate the state of the MNEs during directional motion (Figures S4(b) and (c)). Such normalized areas are the projection areas from the top view, and they are corrected by a coefficient $\cos\alpha$ for comparison to the occupied area when the MNEs are at rest with $\alpha = 0°$. A small normalized average area means that the MNEs are still compact, while a large normalized average area indicates that some MNEs break into fragments, and the smaller fragments or nanomotors may be lost during the migration. MNEs are initially aggregated in the preparation, and their normalized areas show evident periodic area change over time. The average of the normalized area is adopted to estimate the stability of migrating as a group (Figure S4(d)). When the pitch angle is retained as $\alpha \leq 30\,°$, the increase in the normalized average area can be controlled to be less than 25% of the initial state, as shown in the shaded area. Therefore, a small pitch angle is essential for stable directional motion.

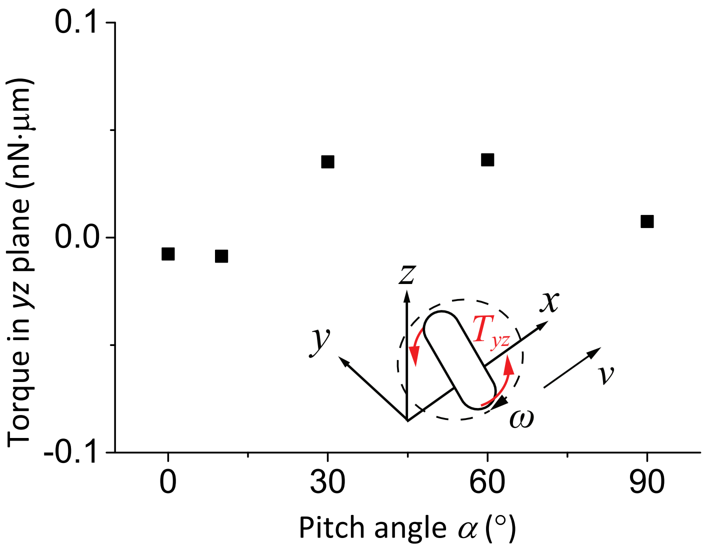

Figure S5: Torque exerted on the MNE in the yz plane as a function of the pitch angle. The inset illustrates the torque T_{yz} in the yz plane .

2 Description of Supporting Movies

Movie S1: Discoloration of the reaction solution with 4-NP degraded by MNEs in a magnetic field and light. The yellow 4-NP is degraded into colorless 4-AP. The magnetic field strength and frequency are $4\,\mathrm{mT}$ and $10\,\mathrm{Hz}$, respectively, and the light power density is $1.5\,\mathrm{W/cm^2}$. The playback speed is 10 times.

Movie S2: Representative motion of MNEs in the catalysis. These five typical kinds of motion are tested in an open tank ($10\,\mathrm{mm} \times 10\,\mathrm{mm} \times 5\,\mathrm{mm}$) with a glass substrate. The movie is recorded by a charge-couple device camera with a frequency of $82\,\mathrm{Hz}$.
